# Deconstructing the Late Phase of Vimentin Assembly by Total Internal Reflection Fluorescence Microscopy (TIRFM)

**DOI:** 10.1371/journal.pone.0019202

**Published:** 2011-04-22

**Authors:** Stefan Winheim, Aaron R. Hieb, Marleen Silbermann, Eva-Maria Surmann, Tatjana Wedig, Harald Herrmann, Jörg Langowski, Norbert Mücke

**Affiliations:** 1 Division Biophysics of Macromolecules, German Cancer Research Center, Heidelberg, Germany; 2 Functional Architecture of the Cell, German Cancer Research Center, Heidelberg, Germany; Dalhousie University, Canada

## Abstract

Quantitative imaging of intermediate filaments (IF) during the advanced phase of the assembly process is technically difficult, since the structures are several µm long and therefore they exceed the field of view of many electron (EM) or atomic force microscopy (AFM) techniques. Thereby quantitative studies become extremely laborious and time-consuming. To overcome these difficulties, we prepared fluorescently labeled vimentin for visualization by total internal reflection fluorescence microscopy (TIRFM). In order to investigate if the labeling influences the assembly properties of the protein, we first determined the association state of unlabeled vimentin mixed with increasing amounts of labeled vimentin under low ionic conditions by analytical ultracentrifugation. We found that *bona fide* tetrameric complexes were formed even when half of the vimentin was labeled. Moreover, we demonstrate by quantitative atomic force microscopy and electron microscopy that the morphology and the assembly properties of filaments were not affected when the fraction of labeled vimentin was below 10%. Using fast frame rates we observed the rapid deposition of fluorescently labeled IFs on glass supports by TIRFM in real time. By tracing their contours, we have calculated the persistence length of long immobilized vimentin IFs to 1 µm, a value that is identical to those determined for shorter unlabeled vimentin. These results indicate that the structural properties of the filaments were not affected significantly by the dye. Furthermore, in order to analyze the late elongation phase, we mixed long filaments containing either Alexa 488- or Alexa 647-labeled vimentin. The ‘patchy’ structure of the filaments obtained unambiguously showed the elongation of long IFs through direct end-to-end annealing of individual filaments.

## Introduction

The shape and the mechanical properties of cells are determined by the cytoskeleton, a network of actin filaments, microtubules and intermediate filaments (IFs), all interconnected by plakins and motor protein complexes [1]. While actin filaments and microtubules have been extensively characterized on the single filament and network level [2]–[6] the physical characterization of IFs has remained limited [7]–[10]. However, the importance of IFs is becoming increasingly recognized, as is evident from current studies, which have demonstrated that mutations within various intermediate filament protein genes may severely affect the mechanical properties of cells and tissues when they are mechanically stressed, thereby leading to tissue-specific diseases [11]–[13].

Filament formation follows three distinct steps and can be initiated by increasing the salt concentration. In the first step tetrameric subunits associate laterally within seconds into so called “unit-length” filaments (ULFs) with a length of 60 nm [14]–[16]. They are assumed to be “minimal filaments” [17]. In the second step all types of filaments, from the “minimal” filament up to long filaments, elongate by longitudinal annealing via end-to-end fusion of their ends. This has been established by extensive mathematic modeling of measured length distributions [17], [18]. During the third phase a radial compaction over time of the open 17-nm-wide filaments to approximately 10-nm-wide mature filaments was observed [19]–[22].

In previous studies, we followed the kinetics of filament formation through filament length profiles of *in vitro* assembled vimentin IFs measured with atomic force microscopy (AFM) and electron microscopy (EM) [17], [18]. For assembly, a phosphate buffer system was used, which enables the dynamic formation of authentic filaments from soluble tetramers by addition of salt to increase the ionic strength to physiological values [16]. In the course of these measurements we found that above a certain length it becomes difficult to trace individual IFs accurately under standard conditions. After extensive dilution, single long filaments can be visualized by AFM in a 40×40 µm scanning field. However, recording significant numbers of filaments is indeed very time consuming. In EM, using image sizes of e.g. 5×5 µm, the number of individual IFs that can be traced is even lower.

In this study, we have generated fluorescently labeled vimentin and characterized the influence the label has on the solubility properties, the assembly kinetics and the lateral compaction of this IF protein by analytical ultracentrifugation, atomic force microscopy and electron microscopy. Using this fluorescent construct, we could observe very long IFs by total internal reflection fluorescence microscopy (TIRFM), allowing us to follow filament growth and to measure IF size with a high rate. Thus, recording the contour of filaments by TIRFM allows rapid determination of their persistence length. The high imaging rate enabled us furthermore to follow the deposition process of IFs in real time. Finally, we were able to reveal the mode of longitudinal IF elongation by observing end-to-end fusion of two different colored vimentin polymers.

## Materials and Methods

### Protein chemical procedures

Recombinant human vimentin was isolated and purified according to published procedures [23]. Labeled and unlabeled vimentin samples were mixed in required ratios (e.g. 10% of labeled filaments) and dialyzed against “*phosphate buffer*” (2 mM sodium phosphate, pH 7.5) by lowering the urea concentration stepwise (8, 6, 4, 2, 1 M). Dialysis was continued overnight at 4°C against fresh *phosphate buffer*. Filament assembly was started by mixing equal volumes of *phosphate buffer* containing 200 mM KCl with a 0.2 g/l vimentin solution at 37°C. The assembly reactions were stopped at defined time points by 1∶10 dilution with *phosphate buffer* containing 100 mM KCl for AFM measurements, 1∶1 for EM measurements, and 1∶500 to 1∶2,500 for TIRFM measurements. For AFM and EM, the pre-diluted samples were further diluted by adding an equal volume of 0.25% glutaraldehyde in *phosphate buffer* containing 100 mM KCl.

### Labeling procedure

Vimentin-containing samples were dialyzed against labeling buffer (5 M urea, 50 mM sodium phosphate, pH 7.0) for 2 hours at room temperature (or over night at 4°C) using dialysis tubing with a molecular weight cut off between 12,000 and 14,000 (Serva, Heidelberg, Germany). Proteins were labeled with Alexa Fluor 488 or 647 maleimide (Invitrogen), solubilized in DMSO at 5 g/l, by adding 1 µl aliquots until a molar ratio of 20∶1 was reached, with brief vortexing after each addition. After 10 to 20 minutes, free reactive dye was captured by the addition of cysteine (1 M in H_2_O) to a concentration of 100 mM and incubated for 1 hour at room temperature with gentle shaking in the dark. The labeled vimentin was separated from free dye by size exclusion chromatography (Bio-Gel P-30; Bio-Rad). A column was prepared using a 5 ml glass pipette (capacity ∼9 ml) filled with about 6 ml of equilibrated resin. 500 µl of labeled sample was applied to the column and washed with labeling buffer. Fractions were tested for vimentin content and purity by conventional SDS-PAGE. Fluorescent molecules were visualized on a Typhoon-Scanner 9400 (GE Healthcare) and band intensities evaluated with ImageJ software (NIH). Peak fractions were pooled, dialyzed for 2 hours at room temperature (or over night at 4°C) against *phosphate buffer* containing 8 M urea and distributed to appropriate aliquots. Samples were stored at −80°C.

### Determination of the labeling efficiency

Protein concentrations were determined by both the Bradford assay (Bio-Rad) with BSA as a standard and absorbance spectroscopy. Absorption spectra were measured on a Cary-4E spectrometer (Varian, Mulgrave, Australia), captured between 220 and 550 nm for Alexa 488 (ε = 71,000 cm^−1^M^−1^) and 220 and 700 nm for Alexa 647 (ε = 237,000 cm^−1^M^−1^) labeled protein and evaluated with the software Cary Win UV (Version 02.00(25)). Dye concentrations were measured with absorbance spectroscopy and fluorescence emission spectroscopy using a SLM-AMINCO 8100 fluorescence spectrometer (SLM, Urbana, IL). Emission spectra were collected between 500 and 650 nm at an excitation wavelength of 495 nm for Alexa 488-labeled samples. The label concentration of the sample was determined based on a standard curve of free dye. The labeling efficiency was calculated as the quotient of measured dye and protein concentration.

### Analytical ultracentrifugation

Analytical ultracentrifugation experiments were carried out in *phosphate buffer* using the Beckman analytical ultracentrifuge (Optima XLA) equipped with an ultraviolet absorption optical system. The sedimentation velocity runs and data analysis were performed as described [16]. Runs were carried out in double-sector charcoal-Epon cells at 20°C and 40,000 rpm. Scans were recorded at 230 nm at a protein concentration of 0.1 g/l using a spacing of 0.003 cm for each data point in a continuous scan mode. Data analysis was done using the program DCDT+ (version 2.2.0) (Philo, 2000).

### Imaging techniques

AFM imaging was performed in air as described [24]. Negatively stained filaments were recorded by EM as described [25]. The diameter of vimentin filaments was measured along individual filaments at intervals of 50 to 100 nm with ImageJ and data were presented as Gaussian curves with the software Origin (7 SR2).

TIRFM imaging was performed on a modified Olympus IX70 microscope fitted with an Olympus TIRFM module, Optosplit dual view (Cairn, Faversham, UK), and recorded with an EMCCD camera (iXon^EM^ + DU-860; Andor, Belfast, N. Ireland) which was controlled using Andor IXon software (Version 4.0). Fluorophores were excited with an argon ion laser at 488 nm (Stabilite 2017; Spectra Physics, Darmstadt, Germany) or a He-Ne Laser at 633 nm (LGK 7626, Lasos Lasertechnik GmbH, Jena, Germany) with exposure times of 50 ms for static images and 5 to 50 ms for dynamic processes. Laser illumination was controlled with an acousto-optic tunable filter (AOTF.nC-VIS-TN 1001; AA OPTO-ELECTRONIC; Orsay, France). An Olympus UPLSAPO 100XO objective was used for TIRFM illumination and imaging, resulting in a resolution of 240 nm/pixel. Filaments were adsorbed onto untreated glass slides (24×60 mm, No. 1 ½, GOLD SEAL®; EMS, Hatfield, PA). To gain knowledge of the mechanical properties of the IFs, images were processed with ImageJ software, tracing the filament backbones manually. The resulting xy-coordinates were used to calculate the length and the persistence length of the filaments as described [24]. Images of two-colored filaments were taken separately by individual laser excitation and further processed by Adobe Photoshop (CS4 extended, v11.0).

## Results and Discussion

### Vimentin labeling with Alexa 488

The visualization by TIRFM offers an excellent opportunity to observe long filaments at short exposure times, however it requires fluorescent labeling of filaments that are not compromised in their biological activity by the labeling agent. Wildtype vimentin of various organisms exhibits one single cysteine residue in the central α-helical rod domain [26]. To obtain specific labeling we decided to apply the chemical label Alexa 488 maleimide, which can be coupled to the cysteine residue. Since this cysteine is located within a highly conserved domain, we considered that a modification with a highly charged conjugated aromatic system as represented by Alexa 488 could easily disturb the complex interaction of vimentin molecules during assembly which is driven both by charge and hydrophobic interactions between the rods and heads [15], [27].

**Figure 1:**
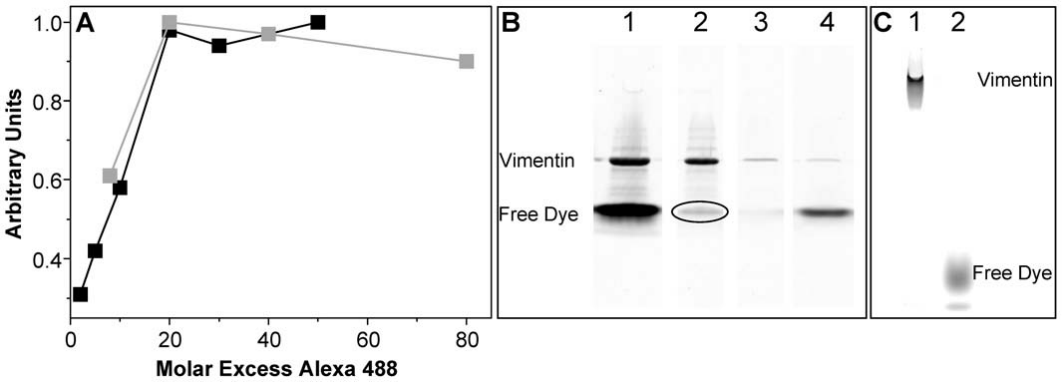
Vimentin can be efficiently fluorescently labeled and purified. (A) Labeling of vimentin at 0.25 g/l (grey) and 1.0 g/l (black) with different Alexa 488 concentrations (2 h incubation at RT). Relative labeling efficiency was determined by applying the samples to a reducing SDS-PAGE and quantifying the intensity of the vimentin band. (B) Reducing SDS-PAGE of relevant fractions indicating the separation of labeled protein from free dye by size exclusion chromatography. (B 1) Vimentin before chromatographic separation; (B 2) Pool of major vimentin fractions; (B 3) Fraction between protein and dye; (B 4) Fraction of free dye. (C) Native PAGE (C 1) shows that free dye band of the major vimentin fraction (B 2) is induced by sample preparation for reducing SDS-PAGE; (C 2) free dye. Protein per lane: (B 2) 1.7 µg and (C 1) 2 µg.

Therefore, we determined the percentage of labeled vimentin that is tolerated before filament formation would be significantly affected. As a prerequisite, we optimized the vimentin labeling procedure using Alexa 488-maleimide. In general, we followed the labeling procedure developed for another IF protein, i.e. lamin A [28], and optimized it with respect to pH, protein concentration and incubation time. In 5 M urea, vimentin forms tetrameric complexes with the single cysteine in the f-position of the coiled coil's heptad repeat optimally positioned for labeling [29], [30]. We used vimentin in concentrations of 0.25 and 1.0 g/l, and found that optimal labeling required a 20 fold molar excess of dye (Figure 1A). Higher dye concentrations did not improve the yield. Furthermore, we investigated the influence of incubation time on the labeling efficiency. According to the supplier (see manual: Thiol-Reactive Probes), proteins should be incubated for 2 hours at room temperature or alternatively for 24 hours at 4°C with a 10–20 fold excess of free dye. However, we found that the labeling reaction is complete within two minutes after the addition of dye.

Free dye was removed from the labeled vimentin by size exclusion chromatography. Effective separation was controlled by SDS-PAGE under reducing conditions (Figure 1B). Only fractions of high vimentin concentration were pooled (Figure 1B, lane 2). Upon evaluation of the SDS gel, the main fraction always exhibited a second faster migrating band corresponding to free dye with approximately 10% of the intensity of the whole lane. However, under native PAGE conditions, no free dye was observed to migrate with the gel front, and fluorescent signal was exclusively found at the position of vimentin (Figure 1C, lane 1). Therefore, the purification by size exclusion chromatography was successful, though a certain amount of dye remained non-covalently bound to vimentin.

**Figure 2:**
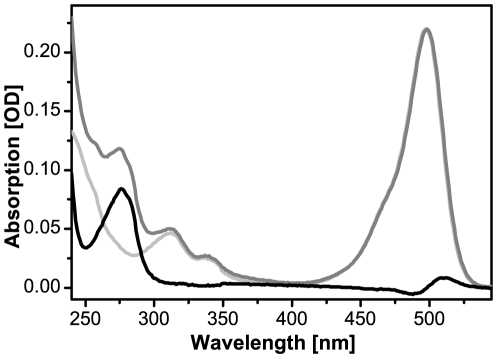
Absorption spectra are used to accurately determine the labeling efficiency of Alexa 488 labeled vimentin. Peaks are evaluated at A_280nm_ for vimentin and at A_495nm_ for the Alexa 488 concentration. To separate the influence of Alexa 488 within the vimentin peak, spectra of pure dye (light grey) are subtracted from the labeled protein spectra (dark grey) to obtain normalized vimentin spectra (black).

We established two independent approaches to quantify the labeling ratios. Firstly, protein and dye concentrations were measured by absorption spectra at 280 and 495 nm, respectively. Due to the fact that Alexa 488 also absorbs in the UV range, overlapping with vimentin, the sample spectrum was corrected by subtracting a spectrum of pure Alexa 488 before evaluating the vimentin peak (Figure 2). Secondly, the vimentin concentration was determined using the Bradford test and dye concentration by fluorescence emission spectra. The results obtained by these methods agreed well, and therefore either method can be used. In the course of various preparations, we reproducibly obtained labeling efficiencies ranging from 60 to 80%.

### Characterization of Alexa 488-labeled starter units and IFs

Labeling of an assembly competent protein may influence its assembly and other biochemical properties. Therefore it is important to investigate the extent to which the presence of a labeled variant is tolerated. On the other hand, for imaging purposes samples must contain sufficient amounts of fluorophore such that TIRFM detection is feasible. To obtain distinct degrees of labeling per filament, samples were prepared at different mixing ratios (10% and 50%) under denaturing conditions (8 M urea) and then dialyzed into phosphate buffer. This procedure should lead to the generation of fluorescently labeled assembly starter units, i.e. tetramers, with different amounts of dyes per complex.

#### Size and shape of assembly starter units by analytical ultracentrifugation

**Figure 3:**
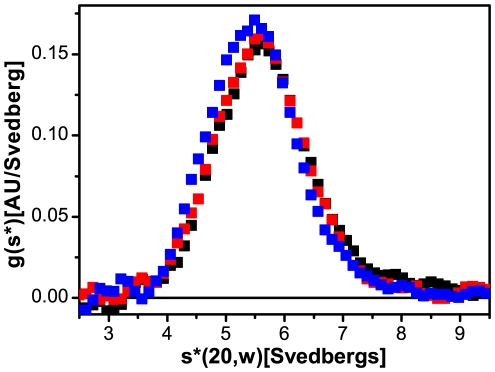
Soluble forms of labeled and unlabeled vimentin have identical characteristics regarding shape, size and homogeneity as revealed by sedimentation velocity analysis (black: unlabeled, red: 10% Alexa 488, blue: 50% Alexa 488).

Sedimentation velocity runs were performed to examine the influence of the fluorescent dye on the size and shape of the soluble complexes (Figure 3). Our data demonstrate that the assembly competent starter units of vimentin sediment as a uniform tetrameric species with an s-value of 5.6 S [16]. With s-values of 5.6 S (10% label) and 5.5 S (50% label) no differences regarding shape and size in between labeled and unlabeled protein were detected. The reported values were the averaged results of two independent runs, whereby the main peaks did not differ more than 0.1 S. The shapes of the sedimentation curves were nearly identical, indicating comparable homogeneity of the soluble pool: we conclude that the modification of vimentin with Alexa 488 has no impact on the solubility of the tetrameric subunits at the examined label concentrations.

#### Longitudinal Assembly

**Figure 4:**
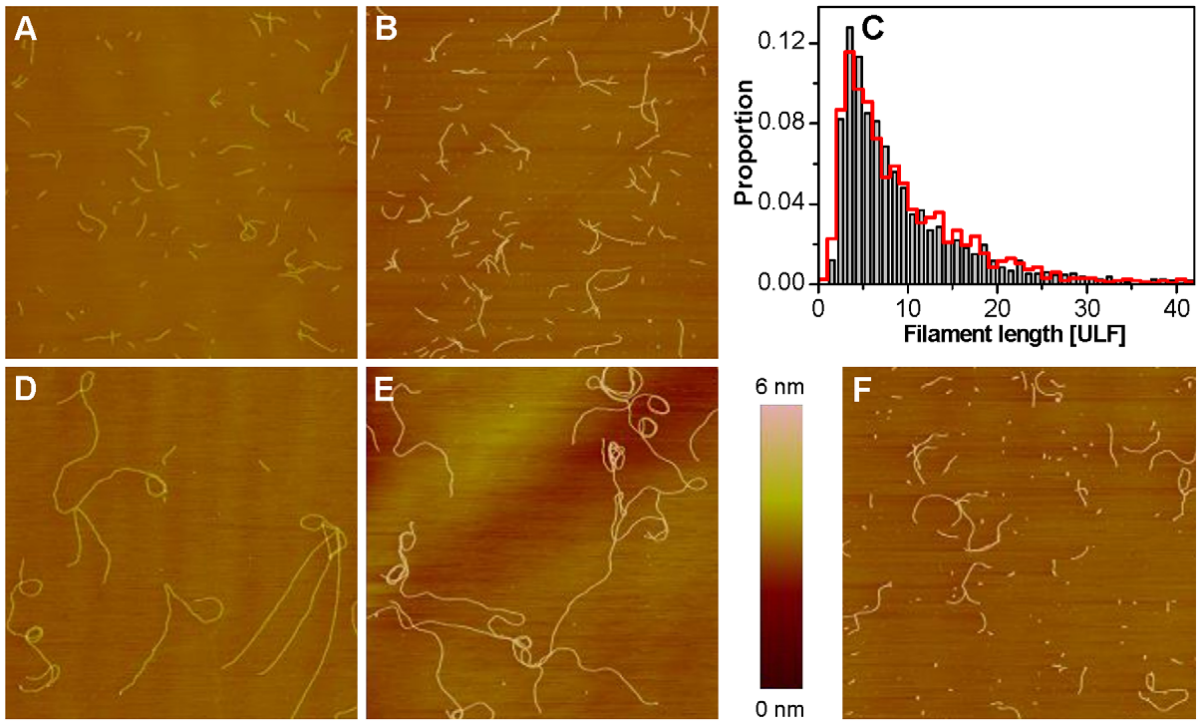
Length distribution of labeled and unlabeled vimentin after defined time points visualized by AFM. After 10 min of assembly vimentin with (A) 0% and (B) 10% of Alexa 488 labeled subunits show comparable assembly kinetics (C); grey: unlabeled (n = 1534); red: 10% label (n = 1645). After an assembly of 1 day, vimentin with (D) 0% and (E) 10% of label show long filaments, in contrast to (F) 50% of label where assembly is clearly affected (Image sizes 10×10 µm).

The influence of Alexa 488-labeling on the longitudinal assembly of vimentin was determined by quantifying the assembly kinetics of the various vimentin mixtures by AFM. IFs were assembled with a protein concentration of 0.1 g/l for 10 minutes at 37°C. For unlabeled and 10% of labeled vimentin, the filaments were smooth and regularly shaped (Figure 4A and B). The quantitative investigation of the length distribution of unlabeled IFs is identical to those of filaments with 10% labeling (Figure 4C), hence clearly demonstrating that these filaments exhibited comparable assembly kinetics. After one day of assembly, long filaments were visible for both unlabeled and 10%-labeled filaments (Figure 4D and E). In parallel, a sample containing 50% of labeled subunits was assembled for one day (Figure 4F). These filaments were shorter and in contrast to the other approaches small fragments were visible that were not incorporated into filaments. This suggests that high levels of protein containing a covalently coupled fluorophore in the coil 2 domain have a significant impact on IF assembly. Hence, for subsequent EM measurements the label amount was kept at 10%.

#### Morphology of labeled filaments

**Figure 5:**
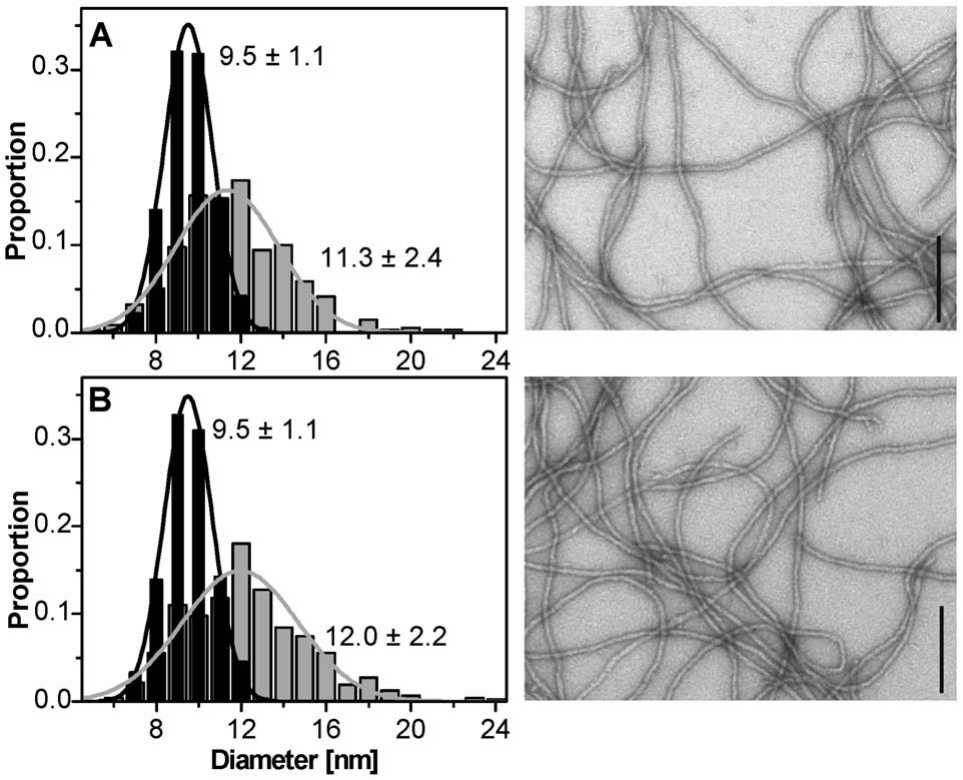
Width measurements of (A) unlabeled vimentin and (B) vimentin with 10% of Alexa 488 labeled subunits. Grey bars: filaments at 10 s of assembly; black bars: filaments at 1 h of assembly (339 to 1656 data points were collected per graph). Filaments are smooth and homogenous as shown by the EM images (bar = 200 nm).

To gain information about the morphology of labeled filaments, vimentin was fixed, negatively stained and visualized with EM after 10 seconds and 1 hour of assembly. After 10 seconds, unlabeled and 10%-labeled filaments were relatively inhomogeneous with diameters of 11.3±2.4 nm and 12.0±2.2 nm, respectively (Figure 5). After an assembly of one hour, smooth, homogeneous filament networks were obtained. The reduction in filament widths to 9.5±1.1 nm was due to the compaction process as previously described for “Tris-buffer” conditions[19]–[21]. Notably, the compaction of filaments over time is much higher in the “Tris-buffer” (about 17 to 10 nm) as compared to the “phosphate-buffer” assembly mode (about 12 to 9.5 nm). Due to the fact that the filament length distribution as well as the diameter of filaments were not affected by the presence of 10% labeled vimentin, subsequent TIRFM assays were performed at this proportion of labeled protein.

### Filament detection and determination of key parameters of IFs with TIRFM

**Figure 6:**
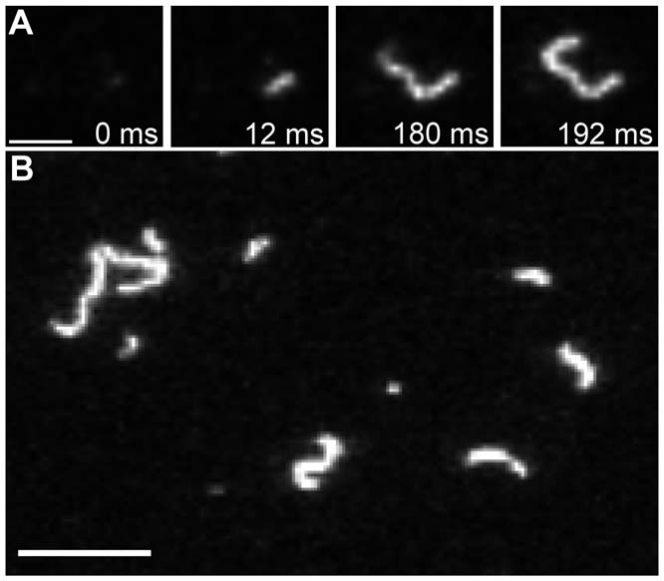
TIRFM images of labeled vimentin. (A) IF adsorption process on a glass surface (bar 2 µm); (B) A single-frame image of vimentin after 240 minutes assembly time (bar 5 µm). Resolution of TIRFM setup is 240 nm per pixel corresponding to 5 to 6 ULFs.

Fluorescently labeled vimentin was assembled for 4 hours, applied to a glass slide and immediately observed by TIRFM. Thus we could record, in real time, the deposition of filaments with different lengths (Figure 6A). This process turned out to be very rapid with deposition times ranging from 20 to 750 ms for 23 observed filaments with a length of 3 to 8 µm.

To monitor the influence of dye on the mechanical properties of vimentin IFs, we have determined the persistence length which is a measure for the apparent flexibility of a polymer chain.

The persistence length represents the statistical relationship between the contour length and the end-to-end distance, and can be calculated for equilibrated filaments as shown by Rivetti ([31],equation 9).

**Figure 7:**
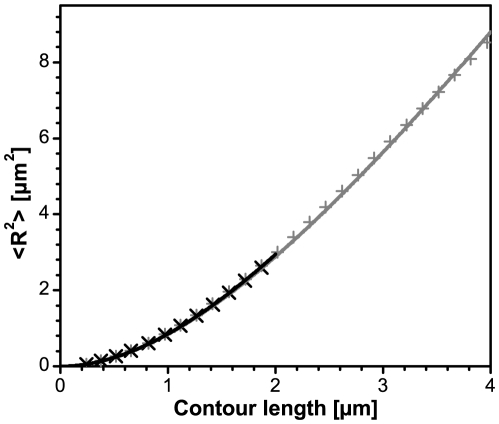
Plot of the mean-square end-to-end distance as a function of the contour length. Nearly identical shapes of the curves indicate that filaments assembled for 2 hours (black, n = 168, 2–4 µm length) and 4 hours (grey, n = 91, 4–15 µm length) have comparable mechanical properties. The data were derived from 10% labeled vimentin equilibrated on glass and visualized with TIRFM.

We therefore traced the contours of filaments adsorbed on glass after 2 h and 4 h of assembly (Figure 6B). The mean-square end-to-end distances were plotted as a function of the contour length, and a persistence length of about 1000 nm for both time points was calculated (Figure 7). This result fits well to data previously obtained for unlabeled vimentin filaments by AFM [24], [32]. We thus have demonstrated that TIRFM is a powerful tool for the analysis and determination of key parameters of long IFs. Moreover, our study confirms TIRFM as a viable method, usable for quantitative purposes, which may complement other established methods such as AFM and EM.

#### Two-color imaging

**Figure 8:**
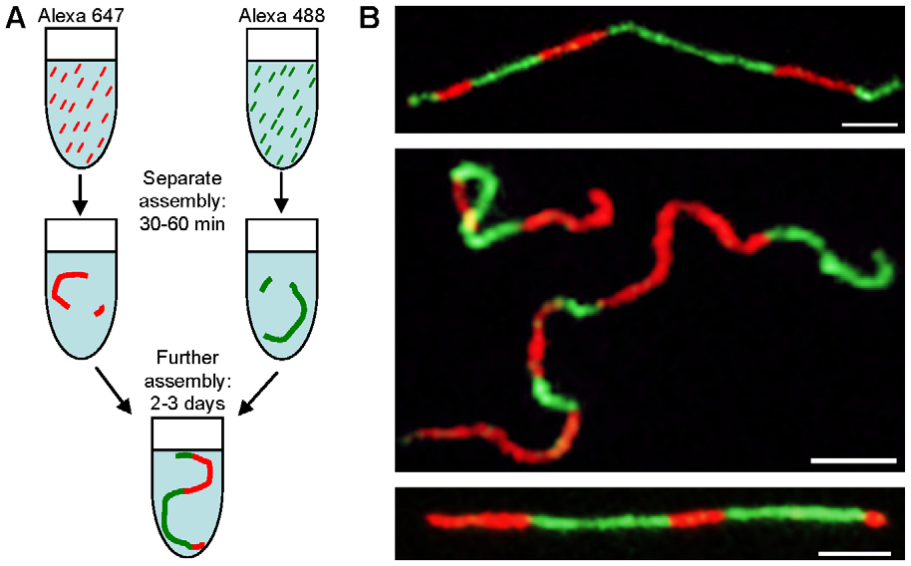
Longitudinal assembly of IFs by end-to-end fusion of filaments. (A) Assembly starter units labeled with two different dyes, assembled separately for 30 min, mixed and further assembled for 2 days. (B) Striped filaments were subsequently observed with TIRFM (bar 3 µm).

In order to visualize the reaction of long filaments in solution, in particular to follow end-to-end assembly of extended filaments directly, we employed vimentin labeled with two distinct fluorophores, i.e. Alexa 488 and Alexa 647. Vimentin preparations containing either 10% of Alexa 488- or Alexa 647-labeled vimentin were first assembled separately for 30 or 60 minutes. Then, both labeled vimentin filament assemblies were mixed and incubated further (Figure 8A). After 2 to 3 days, all filaments observed by TIRFM consisted of red and green segments, indicating that individual filaments of either color had directly annealed end-to-end (Figure 8B). This observation directly demonstrates that the end-to-end fusion of filaments is the principal mode of IF elongation and that dissociation of existing IFs as a source for new assembly is negligible, confirming our previous indirect conclusions from observations of “time-lapse” electron microscopy of ULFs and by mathematically modeling the kinetic parameters of the assembly process [17], [18].

As described the label amount of filaments was 10% which corresponds to the incorporation of 3 monomers per ULFs on average. Based on our calculations about 5% of ULFs have no label, 15% one label, 10% up to 5 labels and 7% have 6 to 8 labels. However, assuming the dye to be stochastically distributed over the filament leads to an apparent inhomogeneity of its brightness and width on fluorescent images. As the filament width was shown to be identical to unlabeled filaments (Figure 5), label amounts of 10% were chosen for an unaffected assembly leading to the stated qualitative conclusions.

### Conclusions

In this study we have optimized the generation of fluorescently labeled vimentin for the unperturbed *in vitro* assembly of filaments. This enabled us to visualize very long fluorescently labeled IFs by TIRFM. The optimized labeling procedure allows us now to design new studies on the co-assembly of vimentin and other IF proteins such as desmin and nestin. Moreover, due to the sensitivity of TIRFM, the high frame rates and large image sizes obtainable, TIRFM in combination with specific labeling of IF proteins will prove highly useful in elucidating the dynamic interaction of IFs with associated proteins.
